# Qualitative and quantitative comparison of image quality between single-shot echo-planar and interleaved multi-shot echo-planar diffusion-weighted imaging in female pelvis

**DOI:** 10.1007/s00330-019-06491-3

**Published:** 2019-12-10

**Authors:** He An, Xiaodong Ma, Ziyi Pan, Hua Guo, Elaine Yuen Phin Lee

**Affiliations:** 1 Department of Diagnostic Radiology, Queen Mary Hospital, University of Hong Kong, Room 406, Block K, Pok Fu Lam Road, Hong Kong, China; 2 Center for Biomedical Imaging Research, Department of Biomedical Engineering, School of Medicine, Tsinghua University, Haidian District, Beijing, China

**Keywords:** Echo-planar imaging, Diffusion magnetic resonance imaging, Female, Pelvis, Artifacts

## Abstract

**Objectives:**

To qualitatively and quantitatively compare the image quality between single-shot echo-planar (SS-EPI) and multi-shot echo-planar (IMS-EPI) diffusion-weighted imaging (DWI) in female pelvis

**Methods:**

This was a prospective study involving 80 females who underwent 3.0T pelvic magnetic resonance imaging (MRI). SS-EPI and IMS-EPI DWI were acquired with 3 *b* values (0, 400, 800 s/mm^2^). Two independent reviewers assessed the overall image quality, artifacts, sharpness, and lesion conspicuity based on a 5-point Likert scale. Regions of interest (ROI) were placed on the endometrium and the gluteus muscles to quantify the signal intensities and apparent diffusion coefficient (ADC). Signal-to-noise ratio (SNR), contrast-to-noise ratio (CNR), and geometric distortion were quantified on both sequences. Inter-rater agreement was assessed using *κ* statistics and Kendall test. Qualitative scores were compared using Wilcoxon signed-rank test and quantitative parameters were compared with paired *t* test and Bland-Altman analysis.

**Results:**

IMS-EPI demonstrated better image quality than SS-EPI for all aspects evaluated (SS-EPI vs. IMS-EPI: overall quality 3.04 vs. 4.17, artifacts 3.09 vs. 3.99, sharpness 2.40 vs. 4.32, lesion conspicuity 3.20 vs. 4.25; *p* < 0.001). Good agreement and correlation were observed between two reviewers (SS-EPI *κ* 0.699, *r* 0.742; IMS-EPI *κ* 0.702, *r* 0.789). IMS-EPI showed lower geometric distortion, SNR, and CNR than SS-EPI (*p* < 0.050). There was no significant difference in the mean ADC between the two sequences.

**Conclusion:**

IMS-EPI showed better image quality with lower geometric distortion without affecting the quantification of ADC, though the SNR and CNR decreased due to post-processing limitations.

**Key Points:**

*• IMS-EPI showed better image quality than SS-EPI.*

*• IMS-EPI showed lower geometric distortion without affecting ADC compared with SS-EPI.*

*• The SNR and CNR of IMS-EPI decreased due to post-processing limitations.*

## Introduction

Magnetic resonance imaging (MRI) is used in the evaluation of malignant and benign diseases of the female pelvis due to its exquisite soft tissue resolution and anatomical details. Diffusion-weighted imaging (DWI) is routinely added as part of the MRI protocol [1–3]. The DWI signal varies according to the tissue microarchitecture or cellularity, reflecting the proportion of intracellular and extracellular water molecules. The log of the slope of the signal decay on DWI is quantified by the apparent diffusion coefficient (ADC), a measure of the diffusion ability of the tissue under investigation [3].

Conventional DWI uses single-shot k-space trajectory echo-planar imaging (SS-EPI), which has the advantage of fast imaging speed and thus, less sensitive to motion [4]. However, SS-EPI can suffer from geometric distortion along tissue boundaries with different susceptibilities since it usually has low bandwidth along the phase-encoding direction [5]. Moreover, SS-EPI has a relatively long readout duration compared with the transverse relaxation time, which can result in blurring artifacts and limit spatial resolution. Therefore, SS-EPI gives rise to low-resolution images and encounters difficulty with large field of view (FOV) [6].

High spatial resolution imaging is important in the assessment of gynecological tumors as a clear and undistorted tumor delineation will allow confident diagnosis and accurate evaluation of the local disease extent. However, peristalsis and air in the gastrointestinal tract and vagina exaggerate the artifacts and the geometric distortion on SS-EPI, hence challenging to achieve high spatial resolution on SS-EPI.

Multi-shot techniques, on the other hand, offer high-resolution DWI by effectively suppressing image distortions. However, they can introduce strong ghost artifact if data are reconstructed directly because of phase variations among different shots [7]. By using phase correction, which is conducted through either extra navigator or self-navigator, the aforementioned artifact can be minimized, and subsequently improves image quality [8–10]. Since the reconstruction with phase correction is basically based on parallel imaging principles, extra navigator is usually needed for high shot numbers such as 6 shots in order to maintain a reliable performance. The multi-shot DWI techniques, including interleaved EPI or readout-segmented EPI [11], have been applied in brain and other body organs with promising results [12–16].

Navigated interleaved multi-shot echo-planar imaging (IMS-EPI) is more effective in distortion reduction compared with readout-segmented EPI [11]. In navigated IMS-EPI, a k-space domain reconstruction method, GRAPPA with a compact kernel is used to recover missing data in each shot, which has been shown to be more robust than image domain phase correction method [10].

Herein, the aims of our study were to compare the image quality and assess the ADC, signal-to-noise ratio (SNR), contrast-to-noise ratio (CNR), and geometric distortion between IMS-EPI and SS-EPI in female pelvis.

## Materials and methods

### Study information

This was a prospective study approved by the local ethics committee with written informed consent from participating subjects. Consecutive females who underwent pelvic MRI in our unit were prospectively recruited between January 2016 and September 2017. Inclusion criteria were (1) females with gynecological symptoms (heavy flow, abnormal bleeding, irregular menses, and dysmenorrhea, etc.); (2) clinical- or ultrasound-suspected uterine congenital anomalies; (3) ultrasound-detected indeterminate masses in the pelvis or with raised CA125; and (4) pre-operative or post-operative assessment of histologic-proven gynecological cancers. Exclusion criteria were those with (1) any contraindications to MRI; (2) hip prosthesis; and (3) no DWI performed.

### MRI technique

All MRI examinations were acquired on a 3T MRI (Achieva 3.0T TX, Philips Healthcare) using a 16-channel phased-array torso coil. All the patients fasted for 6 h and received 20 mg intravenous hyoscine butylbromide (Buscopan, Boehringer Ingelheim) to reduce the peristaltic artifacts. Standard abdominopelvic MRI was performed with the scanning parameters summarized in Table 1. The axial T2-weighted (T2W) images and DWI images had the exact same anatomical coverage, slice thickness, and inter-slice gap to ensure image registration for subsequent analysis.

**Table 1:** Summary of MRI scanning parameters

Sequences	Sagittal T2WI	Coronal T2WI	Axial T2WI	SS-EPI	IMS-EPI	CE 3D T1WI
Pulse	Free-breathing	Free-breathing	Free-breathing	Free-breathing	Free-breathing	Free-breathing
TR/TE (ms)	4654/80	3000/80	2800/100	3240/57	4237/50	3.1/1.45
FOV (mm^2^)	240 × 240	240 × 378	240 × 371	350 × 290	300 × 200	370 × 250
Matrix size	480 × 300	160 × 220	344 × 507	160 × 129	200 × 132	248 × 166
Number of directions	N.A.	N.A.	N.A.	3	3	N.A.
Number of averages	2	1	1	6	2	1
Slice thickness (mm)	4	5	4	4	4	3
WFS (pix)/BW (Hz)	2.002	1.033	2.845	15.156	14.902	0.600
Acquisition time (min)	3.48	2.00	6.90	2.00	6.83	0.32

*CE*, contrast-enhanced; *TR/TE*, repetition time/echo; *FOV*, field of view; *N.A.*, not applicable

SS-EPI and IMS-EPI sequences were acquired using 3 *b* values (*b* = 0, 400, 800 s/mm^2^) based on the same anatomical coverage. The choice of the highest *b* value in this study was based on a balance between sufficient signal suppression of normal tissues in the female pelvis and scan time [17]. The acquisition time for SS-EPI and IMS-EPI was 2.00 min and 6.83 min, respectively. IMS-EPI was acquired using a multi-shot DWI sequence (number of shots = 4), with a partial Fourier factor of 0.76. To be noted, a low-resolution fully sampled navigator was acquired after the image data in each shot for IMS-EPI, which was used for monitoring phase variations and then phase correction in the image reconstruction.

### IMS-EPI reconstruction

**Fig. 1:**
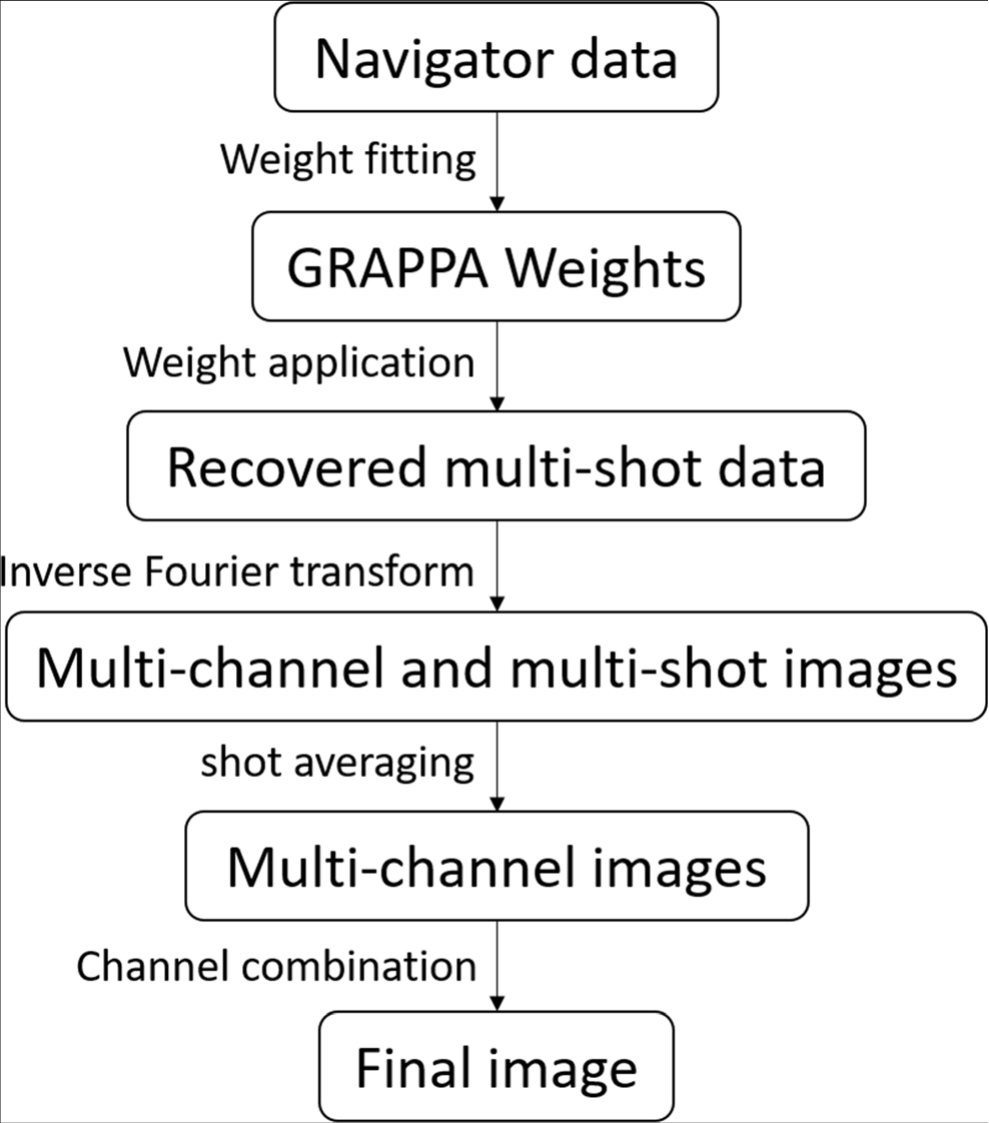
Interleaved multi-shot echo-planar imaging (IMS-EPI) reconstruction method

The image reconstruction was performed in Matlab R2018b (The MathWorks, Inc.). Reconstruction was conducted in the k-space domain using GRAPPA-like interpolation to recover missing data in each shot. Phase variation, which was induced by physiological motion during diffusion gradient encoding, was used for signal encoding, analogy to coil sensitivity encoding. The GRAPPA weights were calibrated from the navigator and applied to the image-echo k-space to recover the data of each channel and shot. The reconstruction method was summarized in Fig. 1. Full details of the reconstruction method were discussed in previous work [10].

### Qualitative assessment

The image quality was assessed on Image J viewing platform 1.45s freeware (National Institutes of Health) by two reviewers (radiologist 1 with 3 years cross-sectional imaging experience; radiologist 2 with more than 10 years cross-sectional and pelvic MRI imaging experience) on separate reading sessions providing independent evaluations. DWI sequences and patients’ sequences were randomly allocated, so the reviewers were blinded to the type of DWI sequences and patient’s clinical information. Qualitative visual assessment was performed on the *b* = 800 s/mm^2^ images and based on a 5-point Likert scale on overall image quality, artifacts, and sharpness. Sharpness was defined by the clarity of the boundaries of the uterus. In patients with an identifiable lesion in the pelvis and without history of pelvic surgery, lesion conspicuity was also assessed (Table 2).

**Table 2:** Image assessment based on the 5-point Likert scale

Score	Overall image quality	Artifacts	Sharpness	Lesion conspicuity
1	Non-diagnostic	Non-diagnostic	Non-diagnostic	Lesion unidentifiable
2	Substantial deficits in image quality	Substantial impact on diagnosis	Not sharp	No differentiation between lesion and normal anatomy
3	Moderate image quality	Moderate impact on diagnosis	A little sharp	Subtle lesion with poorly defined edges
4	Good image quality	Little impact on image diagnosis	Moderately sharp	Well-seen lesion with poorly defined edges
5	Excellent image quality	No artifact	Satisfying sharp	Well-seen lesion with well-defined edges

### Quantitative assessment

#### ADC

Patients with history of hysterectomy were excluded from the quantitative assessment. ADC maps were generated from both DWI sequences with a mono-exponential fit based on the acquired 3 *b* values using in-house scripts written on MATLAB.

**Fig. 2:**
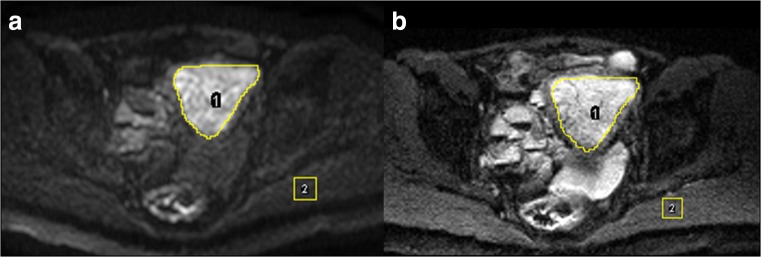
ROIs on the (1) endometrium and (2) gluteus muscles on *b* = 800 s/mm^2^ images SS-EPI (**a**) and IMS-EPI (**b**)

Radiologist 1 placed two sets of regions of interest (ROIs) on *b* = 800 s/mm^2^ SS-EPI and IMS-EPI (Fig. 2) and then transferred to the corresponding ADC maps with reference to the T2W images. ROI 1 was placed in the endometrium on the slice with the largest diameter and ROI 2 (1 cm × 1 cm) was placed in the gluteus muscles (GM) on the same slice to quantify the ADC values.

#### Signal-to-noise ratio and contrast-to-noise ratio

The aforementioned ROIs were also transferred to *b* = 0 and 400 s/mm^2^ images. The average signal within the ROIs in the endometrium and GM on different *b* values was denoted as S_ENDO0_, S_ENDO400_, S_ENDO800_, S_GM0_, S_GM400_, and S_GM800_, respectively. Signal-to-noise ratio (SNR) was defined as the signal of endometrium divided by the standard deviation [18]:

$$ \mathrm{SNR}={\mathrm{S}}_{\mathrm{ENDO}}/{\mathrm{S}\mathrm{D}}_{\mathrm{ENDO}} $$

Contrast-to-noise ratio (CNR) was defined as the absolute signal difference of endometrium and GM divided by the standard deviation of GM [18]:

$$ \mathrm{CNR}=\mid {\mathrm{S}}_{\mathrm{ENDO}}-{\mathrm{S}}_{\mathrm{GM}}\mid /{\mathrm{S}\mathrm{D}}_{\mathrm{GM}} $$

#### Geometric distortion

This was assessed by measuring the deviations in maximal diameter of the uterus in transverse and anterior-posterior directions between the two DWI sequences on the slice that the uterus appeared largest. The measurements on T2W images on the correlated slice were taken as standard of reference.

### Statistical analysis

Inter-rater agreement for qualitative image quality was assessed using *κ* statistics (< 0, poor; 0.01–0.20, slight; 0.21–0.40, fair; 0.41–0.60, moderate; 0.61–0.80, good; 0.81–0.99, almost perfect) [19]. The correlation between the reviewers’ scores was determined by Kendall test. Qualitative scores were compared using Wilcoxon signed-rank test; the mean ADC, SNR, CNR, and geometric distortion between SS-EPI and IMS-EPI were compared using paired *t* test and Bland-Altman analysis after testing for normality. All statistical analyses were performed using SPSS software (version 22.0, SPSS Inc.). *P* < 0.05 was considered as statistically significant.

## Results

### Demographics

Eighty patients (mean age 53.9, range 23–86 years old) were included in the qualitative assessment. The indications for pelvic MRI included (1) for investigation of gynecological symptoms (heavy flow, abnormal bleeding, irregular menses, and dysmenorrhea etc.) (*n =* 29); (2) clinical- or ultrasound-suspected uterine congenital anomalies (*n* = 2); (3) ultrasound-detected indeterminate masses in the pelvis or with raised CA125 (*n =* 15); and (4) pre-operative or post-operative assessment of histologic-proven gynecological cancers (*n =* 34).

Twelve patients had hysterectomy previously, thus were excluded from subsequent quantitative analysis. Among the 68 patients who were included in the quantitative analysis, there were endometrial cancer (*n =* 37), benign diseases (uterine fibroid, *n =* 12; adenomyosis, *n* = 3; ovarian teratoma; *n* = 2), uterine congenital anomalies (*n =* 2), other gynecological malignancies (cervix carcinoma, *n =* 3; ovarian carcinoma, *n =* 3; carcinoma of vulva, *n =* 2), and 4 cases with no structural abnormality found.

### Qualitative assessment

**Fig. 3:**
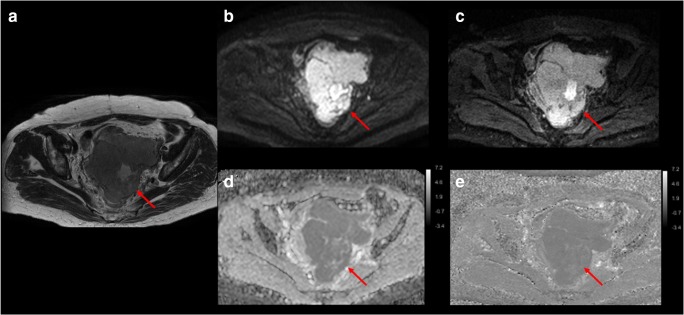
An example of single-shot k-space trajectory echo-planar imaging (SS-EPI) and interleaved multi-shot echo-planar imaging (IMS-EPI) of a patient with endometrial cancer (red arrow). **a** Axial T2WI. **b** SS-EPI (*b* = 800 s/mm^2^). **c** IMS-EPI (*b* = 800 s/mm^2^). **d** ADC map for SS-EPI. **e** ADC map for IMS-EPI

**Fig. 4:**
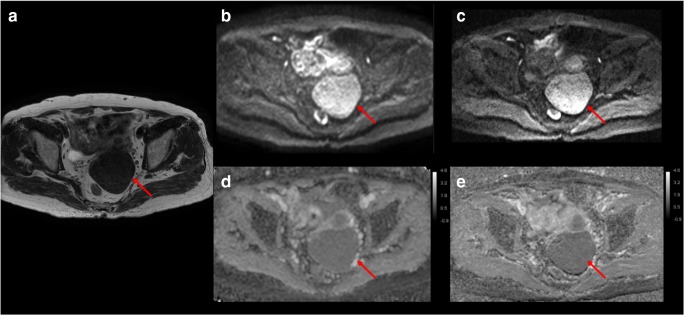
An example of single-shot k-space trajectory echo-planar imaging (SS-EPI) and interleaved multi-shot echo-planar imaging (IMS-EPI) of a patient with leiomyoma (red arrow). **a** Axial T2WI. **b** SS-EPI (*b* = 800 s/mm^2^). **c** IMS-EPI (*b* = 800 s/mm^2^). **d** ADC map for SS-EPI. **e** ADC map for IMS-EPI

**Fig. 5:**
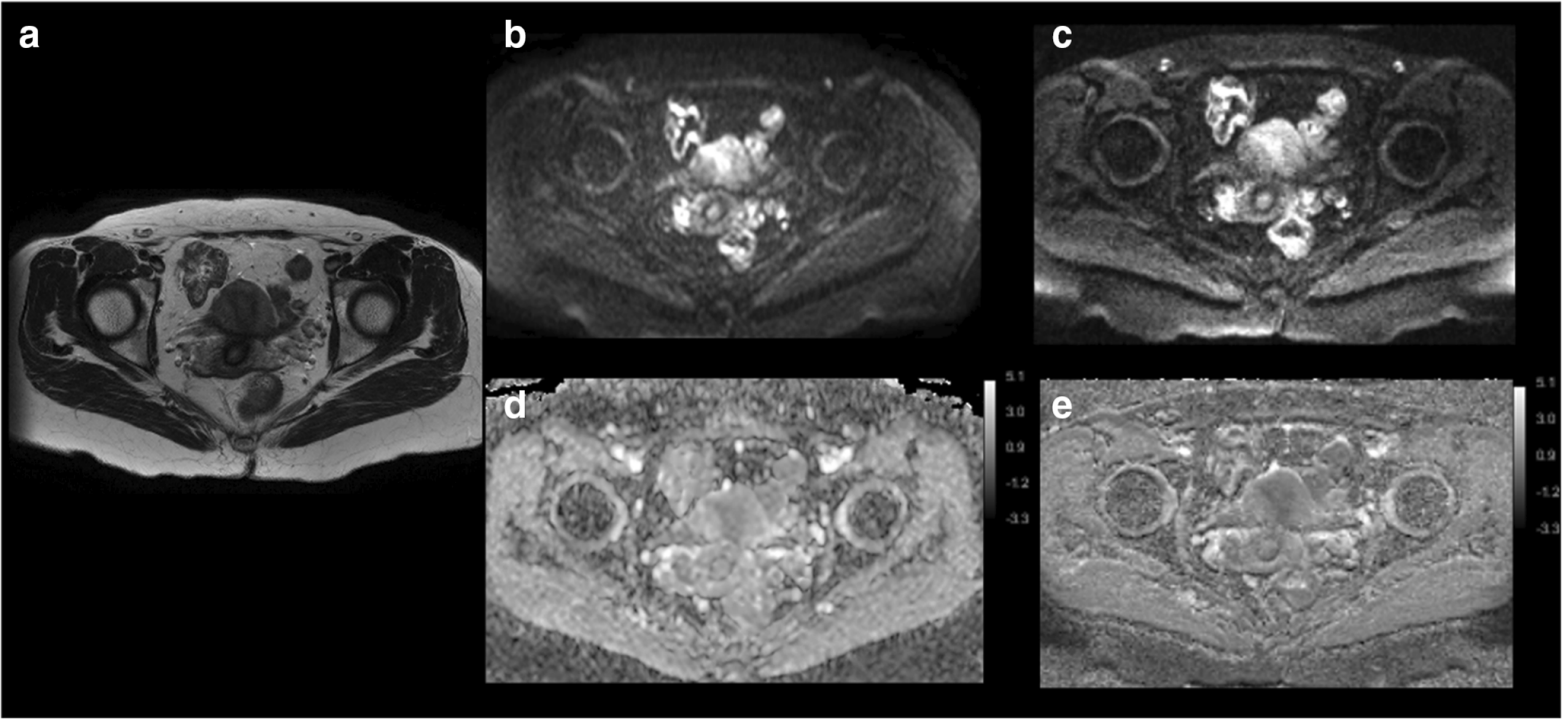
An example of single-shot k-space trajectory echo-planar imaging (SS-EPI) and interleaved multi-shot echo-planar imaging (IMS-EPI) of patient without gynecological abnormality. **a** Axial T2WI. **b** SS-EPI (*b* = 800 s/mm^2^). **c** IMS-EPI (*b* = 800 s/mm^2^). **d** ADC map for SS-EPI. **e** ADC map for IMS-EPI

IMS-EPI scored higher image quality than SS-EPI on all the qualitative factors evaluated regardless of the experience of reviewers (Figs. 3, 4, and 5; Table 3). The average scores between the reviewers for SS-EPI vs. IMS-EPI were as follows: overall quality 3.04 vs. 4.17, artifacts 3.09 vs. 3.99, sharpness 2.40 vs. 4.32, and lesion conspicuity 3.20 vs. 4.25 (*p* < 0.001).

**Table 3:** Qualitative scores between the two reviewers

	Overall image	Artifacts	Sharpness	Lesion conspicuity
*N*	80	80	68	62
Reader 1				
SS-EPI	3.15	3.34	2.41	3.13
IMS-EPI	4.20	4.09	4.29	4.24
*p*	< 0.001	< 0.001	< 0.001	< 0.001
Reader 2				
SS-EPI	2.94	2.98	2.40	3.29
IMS-EPI	4.13	3.95	4.34	4.26
*p*	< 0.001	< 0.001	< 0.001	< 0.001

*SS-EPI*, single-shot k-space trajectory echo-planar imaging (SS-EPI); *IMS-EPI*, multi-shot echo-planar imaging

The median *κ* scores between the two reviewers were high: SS-EPI 0.699 (95% confidence interval (CI), 0.630–0.768) and IMS-EPI 0.702 (95% CI, 0.631–0.773) [19]. The average Kendall *r* correlations were significantly positive (SS-EPI: *r* = 0.742, *p* < 0.001; IMS-EPI: *r* = 0.789, *p* < 0.001).

### Quantitative assessment

**Fig. 6:**
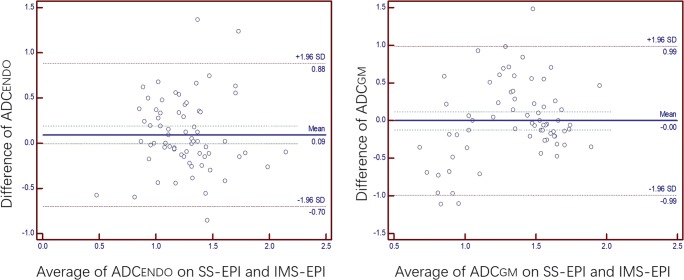
Bland-Altman plots comparing the ADC values between single-shot k-space trajectory echo-planar imaging (SS-EPI) DWI and interleaved multi-shot echo-planar imaging (IMS-EPI) DWI. ADC_GM_: the average ADCs of gluteus muscle; ADC_ENDO_: the average ADCs of endometrium

The average ADCs of endometrium were (1.307 ± 0.354) × 10^−3^ mm^2^/s on SS-EPI and (1.214 ± 0.348) × 10^−3^ mm^2^/s on IMS-EPI (*p* = 0.063). The average ADCs of GM were (1.348 ± 0.454) × 10^−3^ mm^2^/s on SS-EPI and (1.349 ± 0.343) × 10^−3^ mm^2^/s on IMS-EPI (*p* = 0.976) (Fig. 6).

The average SNR of SS-EPI was 5.305 ± 1.803, 5.695 ± 2.213, and 5.465 ± 1.978 in comparison to that of 4.539 ± 1.693, 4.752 ± 1.915, and 5.004 ± 2.198 on IMS-EPI on *b* = 0, 400, and 800 s/mm^2^ (*p* < 0.001, *p* < 0.001, and *p* = 0.030), respectively. The average CNR of SS-EPI was 11.406 ± 6.205, 13.816 ± 7.134, and 14.122 ± 8.613 in comparison to that of 7.414 ± 8.622, 6.504 ± 6.058, and 7.183 ± 5.331 on IMS-EPI on *b* = 0, 400, and 800 s/mm^2^ (*p* < 0.001, *p* < 0.001, and *p* < 0.001), respectively.

The average geometric distortion on SS-EPI was 4.093 ± 3.336 mm (transverse direction) and 3.062 ± 3.680 mm (anterior-posterior direction) and on IMS-EPI was 3.180 ± 2.306 mm (transverse direction) and 2.377 ± 2.068 mm (anterior-posterior direction) (*p* = 0.044, 0.018), respectively.

## Discussion

In this study, we showed that IMS-EPI had superior image quality and decrease geometric distortion compared with SS-EPI without affecting ADC quantification. However, the SNR and CNR suffered, being lower on IMS-EPI.

Traditionally, DWI based on SS-EPI allows rapid acquisition but unfortunately is more susceptible to artifacts, such as chemical shift, Nyquist ghost, image blurring, and geometric distortion. By lowering FOV in the phase-encoding direction of the EPI read-out, the off-resonance-induced artifact could be minimized and image quality could be improved [20]. With zoomed EPI, instead of standard EPI pulse, the distortion and ghosting artifacts can be reduced. [21, 22] However, for patients with disseminate diseases, a reduced FOV cannot fulfill the clinical need for whole pelvic evaluation. Moreover, the mean tumor ADC obtained with zoomed EPI is not stable [23–25].

The image quality was improved on IMS-EPI with superior overall image quality, less artifacts, increased sharpness of the image, and higher lesion conspicuity when compared with SS-EPI. The findings were consistent between reviewers, regardless of their experience with substantial inter-rater agreement. In comparison with SS-EPI, IMS-EPI has higher bandwidth in phase-encoding direction, which can reduce distortion and improve spatial resolution, thus providing higher fidelity for the image details, explaining the improved image quality on IMS-EPI.

The ADC has been shown to be a strong predictor for histologic subtype and tumor grade in endometrial cancer [26] but can be influenced by magnetic field strength, sequence protocols, and the *b* values used [27]. The novel reconstruction method used by IMS-EPI did not affect the quantification of ADC. In other words, quantification of IMS-EPI is at least as robust as SS-EPI with benefit of better image quality. This is important as the absolute value of ADC can be used to differentiate malignant endometrial lesion from normal endometrium [28]. Furthermore, the change in ADC can monitor treatment response and it is imperative that the derivation of ADC is reproducible, in order to trace the real therapeutic magnitude [29].

Usually, normal myometrium was taken as reference when assessing CNR in female pelvis [30], but in our study, many of the patients with endometrial cancer had bulky tumors and the identification of normal myometrium was inconsistent and unreliable. For some patients with diffuse adenomyosis, the identification of normal myometrium on the same slice as the endometrium ROI was also challenging. As such, we had chosen to take GM signal as an alternative reference [18].

We observed lower SNR and CNR on IMS-EPI than SS-EPI, which could be a result from the differences in spatial resolution and post-processing method. Given that IMS-EPI offered higher spatial resolution, the SNR would decrease unless more averages were used. Nevertheless, increasing averages would incur penalty in the scan time and make this unpractical for clinical use. Therefore, in this study, we elected to use two as a trade-off between SNR and scan time. The CNR drop in IMS-EPI is probably caused by the enhanced signal intensity in the GM (Fig. 2) due to coil sensitivities, but we were not able to conduct the uniformity correction like traditional DWI since the IMS-EPI was reconstructed offline. Furthermore, the differences in imaging parameters of both sequences could account for the signal differences in the tissues investigated.

Nevertheless, despite lower SNR and CNR, the overall image quality was higher on IMS-EPI, likely attributed to the significant improvement in geometric distortion on IMS-EPI and hence confidence in defining anatomical borders. Studies have shown that DWI coupled with T2W images can improve the evaluation of various gynecological cancers. A prediction model was constructed combining both sequences to evaluate parametrial invasion (PMI) in cervical cancer [31]; DWI significantly increases the specificity of MR imaging in the detection of residual tumor compared with T2W images alone in cervical cancer after radiotherapy [32, 33]. For endometrial cancer, the depth of myometrial invasion and the presence of lymph node metastasis are important prognostic factors. DWI coupled with T2W images can offer high diagnostic performance with an area under the receiver operating characteristic (ROC) curve of 0.94 in predicting myometrial invasion [34]. The reduction in geometric distortion on IMS-EPI would benefit the co-registration between the DWI and anatomical images and potentially allow more accurate assessment, better surgical planning and treatment stratification.

However, the current longer scan time incurred in IMS-EPI would limit its clinical utility but other techniques could be considered to improve the acquisition efficiency, for example, the application of simultaneous multi-slice technique, which could shorten scan time by a factor of 2–3 without losing significant SNR [12, 35]. Furthermore, the development of an on-line image reconstruction would also improve the clinical acceptance of this promising technique.

Our study has limitations. First, we had a heterogeneous cohort of patients with various pelvic conditions including both malignant and benign diseases; thus, the merits of IMS-EPI in assisting the diagnosis of specific disease such as endometrial cancer could not be evaluated. Second, the inherent longer scan time required by IMS-EPI and offline image reconstruction may limit its clinical translation currently. Continual effort is needed to improve the efficiency of data acquisition and a more streamline post-processing algorithm to minimize these hindrances of a promising technique. For example, simultaneous multi-slice technique can be used to accelerate IMS-EPI DWI [35, 36], with a smaller decrease of SNR compared with traditional parallel imaging techniques.

In conclusion, IMS-EPI showed higher image quality and lower geometric distortion compared with SS-EPI without affecting the mean ADC, potentially a promising technique in improving assessment in female pelvis. However, the SNR and CNR suffered due to the post-processing limitations.

## Abbreviations and acronyms

ADC: Apparent diffusion coefficient
CNR: Contrast-to-noise ratio
DWI: Diffusion-weighted imaging
FOV: Field of view
GM: Gluteus muscles
IMS-EPI: Interleaved multi-shot echo-planar imaging
MRI: Magnetic resonance imaging
PMI: Parametrial invasion
ROC: Receiver operating characteristic
ROI: Regions of interest
SNR: Signal-to-noise ratio
SS-EPI: Single-shot k-space trajectory echo-planar imaging
